# 周期性血小板减少症一例

**DOI:** 10.3760/cma.j.issn.0253-2727.2021.09.012

**Published:** 2021-09

**Authors:** 冰洁 丁, 柳 刘, 梦娟 李, 敖 夏, 雪雯 宋, 可树 周, 健 周, 佩佩 徐, 建平 刘, 虎 周, 永平 宋

**Affiliations:** 1 郑州大学附属肿瘤医院、河南省肿瘤医院血液科 450008 Department of Hematology, Affiliated Cancer Hospital of Zhengzhou University, Henan Cancer Hospital, Zhengzhou 450008, China; 2 郑州大学第一附属医院血液科 450052 Department of Hematology, First Affiliated Hospital of Zhengzhou University, Zhengzhou 450052, China

患者，男，42岁，于2020年8月以“发现血小板减少伴皮肤瘀点3年余”入院。2017年7月患者无明显诱因出现皮肤散在出血点，偶有齿龈出血、血便，未予重视。2019年6月初因皮肤瘀点增多到当地县医院就诊，查血常规示WBC 7.48×10^9^/L、HGB 158 g/L、PLT 10×10^9^/L，给予地塞米松10 mg/d×3 d治疗，血小板计数升至26×10^9^/L。2019年6月底血小板计数6×10^9^/L，骨髓涂片：骨髓有核细胞增生活跃，粒红系比例形态正常，全片见巨核细胞123个，分类25个，其中幼稚巨核细胞4个，颗粒巨核细胞20个，产板巨核细胞1个，血小板少见，形态未见明显异常。当地诊断为“原发免疫性血小板减少症”，给予对症支持及输血治疗，血小板计数升至251×10^9^/L。出院1个月后再次出现皮肤瘀点，复查血小板计数为15×10^9^/L，给予“糖皮质激素”治疗及血小板输注，血小板计数恢复正常水平。后多次复查血常规发现，每月10日左右四肢出现皮肤瘀点，4～5 d后血小板计数降至20×10^9^/L以下，17日复查血小板升至30×10^9^/L以上，此后逐渐升高（峰值达450×10^9^/L）。近两月患者自行服用中成药治疗，血小板计数仍按上述规律周期变化。既往史：2019年幽门螺杆菌感染，未服用药物，后复查阴性。入院查体：全身皮肤黏膜无瘀点及瘀斑，胸骨无压痛，心肺听诊无异常。血常规：WBC 7.79×10^9^/L，HGB 160 g/L，PLT 392×10^9^/L；骨髓涂片：骨髓增生明显活跃，粒红系比例形态大致正常，全片见巨核细胞2210个，分类25个，其中5个产板型，20个颗粒型；血小板散在/成簇易见。骨髓活检：骨髓增生大致正常（60％～70％），粒红比例略减少，巨核细胞易见。网状纤维染色（MF-0级）。小巨核酶标：全片巨核细胞770个，数量增多，其中正常巨核细胞（胞体>40 µm）740个，双核巨核细胞（胞体>40 µm）27个，多核巨核细胞（胞体>40 µm）3个。血小板自身抗体六项：血小板放散液GPⅡb/Ⅲa（−），血小板放散液GPⅠa/Ⅱa（−），血小板放散液GPⅠb/Ⅸ（−），循环血浆GPⅡb/Ⅲa（−），循环血浆GPⅠb/Ⅸ（−），循环血浆Ⅰa/Ⅱa（−）。大颗粒淋巴细胞免疫分析：CD3+T淋巴细胞占淋巴细胞的92.17％（参考值64％～76％）。淋巴细胞亚群绝对计数：CD3^+^细胞91.7％（参考值60.0％～84.0％），CD3^+^CD8^+^细胞44.9％（参考值20.0％～35.0％），CD3^+^CD8^+^细胞计数1341/µl（参考值360～1250/µl）。甲功5项（FT3、FT4、TSH、TG、AnTi-Tg）正常。抗核抗体谱阴性。在院期间隔日复查1次血常规，观察一个周期血小板计数变化与患者自诉规律相符，血红蛋白及白细胞无周期性波动。最终诊断为周期性血小板减少症。给予环孢素A 200 mg/d口服，血小板计数低于30×10^9^/L时给予泼尼松60 mg/d，3 d后减为30 mg/d，逐渐减量至血小板计数高于30×10^9^/L停用。血小板计数高于350×10^9^/L时给予阿司匹林肠溶片100 mg/d治疗。

